# Di-μ-bromido-bis­{[*N*,*N*-dimethyl-*N*′-(thio­phen-2-yl­methyl­idene)ethane-1,2-diamine]­copper(I)]}

**DOI:** 10.1107/S1600536812017989

**Published:** 2012-04-28

**Authors:** Christopher Goh, Zachary D. Remillard, Andre P. Martinez, Amanda C. Keeley, Jerry P. Jasinski

**Affiliations:** a Department of Chemistry, Williams College, Williamstown, MA 01267, USA; b Department of Chemistry, Keene State College, 229 Main Street, Keene, NH 03435-2001, USA

## Abstract

In the crystal structure of the title compound, [Cu_2_Br_2_(C_9_H_14_N_2_S)_2_], the mol­ecule resides about a crystallographic inversion center. The coordination sphere around each copper ion has a distorted tetra­hedral geometry, with ligation by two bridging bromide ions, an amine N atom and an imine N atom. The thio­phene ring is disordered over two sites, with occupancies of 0.719 (3) and 0.281 (3). Weak C—H⋯π inter­actions feature in the crystal packing.

## Related literature

For catalysts for polymerizations and organic transformations, see: Perrier *et al.* (2002 ▶), Cristau *et al.* (2005 ▶). For model complexes of copper proteins, see: Lee *et al.* (2010 ▶). For metal-mediated atom-transfer radical polymerizations, see: Matyjaszewski & Tsarevsky (2009 ▶). For related structures with a Cu_2_Br_2_ core, see Ball *et al.* (2001 ▶), Skelton *et al.* (1991 ▶), Churchill *et al.* (1984 ▶). For software for searching the Cambridge Structural Database, see: Bruno *et al.* (2002 ▶). For standard bond lengths, see Allen *et al.* (1987 ▶).
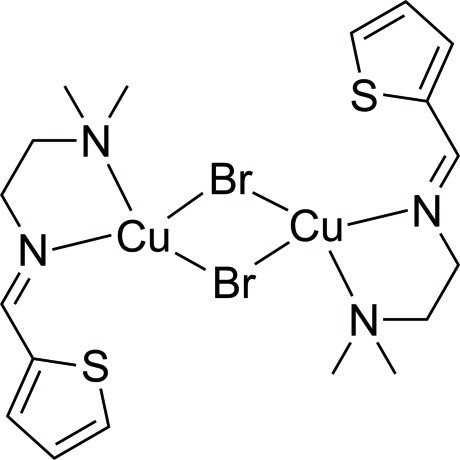


## Experimental

### 

#### Crystal data

[Cu_2_Br_2_(C_9_H_14_N_2_S)_2_]
*M* *_r_* = 651.48
Monoclinic, 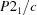

*a* = 10.2029 (3) Å
*b* = 15.4175 (3) Å
*c* = 8.04875 (19) Å
β = 108.628 (3)°
*V* = 1199.76 (5) Å^3^
*Z* = 2
Mo *K*α radiation
μ = 5.29 mm^−1^
*T* = 173 K
0.15 × 0.07 × 0.05 mm

#### Data collection

Oxford Diffraction Xcalibur Eos Gemini diffractometer
Absorption correction: multi-scan (*CrysAlis PRO*; Oxford Diffraction, 2010 ▶) *T*_min_ = 0.406, *T*_max_ = 1.000
13662 measured reflections
3928 independent reflections
3021 reflections with *I* > 2σ(*I*)
*R*_int_ = 0.046

#### Refinement

*R*[*F* ^2^ > 2σ(*F* ^2^)] = 0.036
*wR*(*F* ^2^) = 0.078
*S* = 1.05
3928 reflections
146 parameters
10 restraints
H-atom parameters constrained
Δρ_max_ = 0.50 e Å^−3^
Δρ_min_ = −0.51 e Å^−3^

### 

Data collection: *CrysAlis PRO* (Oxford Diffraction, 2010 ▶); cell refinement: *CrysAlis PRO*; data reduction: *CrysAlis RED* (Oxford Diffraction, 2010 ▶); program(s) used to solve structure: *SHELXS97* (Sheldrick, 2008 ▶); program(s) used to refine structure: *SHELXL97* (Sheldrick, 2008 ▶); molecular graphics: *SHELXTL* (Sheldrick, 2008 ▶); software used to prepare material for publication: *SHELXTL*.

## Supplementary Material

Crystal structure: contains datablock(s) global, I. DOI: 10.1107/S1600536812017989/fj2540sup1.cif

Structure factors: contains datablock(s) I. DOI: 10.1107/S1600536812017989/fj2540Isup2.hkl

Additional supplementary materials:  crystallographic information; 3D view; checkCIF report

## Figures and Tables

**Table 1:** Hydrogen-bond geometry (Å, °) *Cg*1, *Cg*3 and *Cg*4 are the centroids of the Br1/Cu1/Br1*A*/Cu1*A*, S1*A*/C1*A*/C2*A*/C3*A*/C4*A* and S1*B*/C1*B*/C2*B*/C3*B*/C4*B* rings, respectively.

*D*—H⋯*A*	*D*—H	H⋯*A*	*D*⋯*A*	*D*—H⋯*A*
C2*A*—H2*AA*⋯*Cg*3^i^	0.93	2.87	3.721 (8)	153
C2*A*—H2*AA*⋯*Cg*4^i^	0.93	2.70	3.573 (12)	157
C2*B*—H2*BA*⋯*Cg*1	0.93	2.55	3.45 (2)	162
C2*B*—H2*BA*⋯*Cg*1^ii^	0.93	2.55	3.45 (2)	162

Symmetry codes: (i) 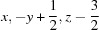; (ii) 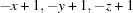.

**Fig. 1.**
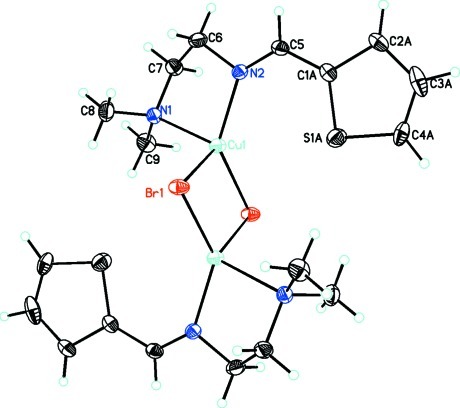
Molecular structure of the title compound (I) showing the atom labeling scheme of the asymmetric unit and 30% probability displacement ellipsoids. A crystallographic inversion center generates the complete molecule. Only the major component (S1A/C1A/C2A/C3A/C4A) of the disordered thiophene ring (occupancy: 0.719) is displayed.

**Fig. 2.**
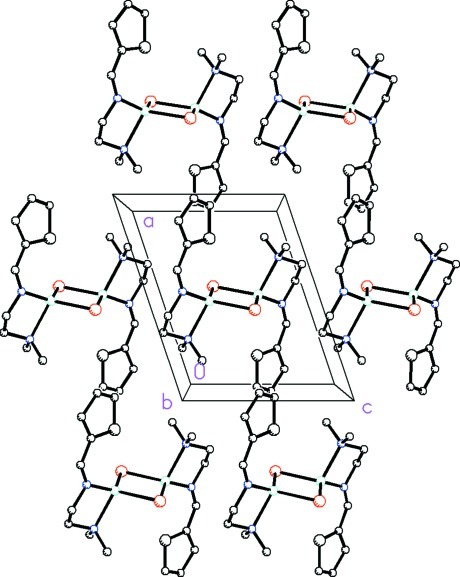
Packing diagram of the title compound viewed along the b axis. Only the major component (S1A/C1A/C2A/C3A/C4A) of the disordered thiophene ring (occupancy: 0.719) is displayed. The hydrogen atoms have been removed for clarity.

## supplementary crystallographic information

### Comment

Copper complexes of ligands containing hetero-aromatic and amine donor moieties have found multiple applications in metal catalyzed processes. Examples include catalysts for polymerizations and organic transformations (Perrier *et al.*, 2002; Cristau *et al.*, 2005), and model complexes in the biomimetic study of copper proteins (Lee *et al.*, 2010). Our group has been interested in the use of neutral tridentate hetero-aromatic-amine ligands in metal-mediated atom transfer radical polymerizations (ATRP) (Matyjaszewski & Tsarevsky, 2009).

Here we report the synthesis and structure of a doubly bromide bridged dinuclear copper(I) complex with the ligand *N*,*N*-dimethyl-*N*'-(thiophen-2-ylmethylene)ethane-1,2-diamine, [{(C_4_H_3_S)CHNCH_2_CH_2_N(CH_3_)_2_}CuBr]_2_ (Fig. 1). A crystallographic inversion center generates the complete molecule from the asymmeric unit. The coordination sphere around each copper ion is arranged in a distorted tetrahedral geometry, with ligation by two bridging bromide ions, an amine nitrogen and an imine nitrogen. The thiophene ring is disordered (occupancy 0.719:0.281). The distances for the metal-amine bond (2.008 (2) Å) and the metal-imine bond (2.240 (2) Å) are within expected ranges (Allen *et al.*, 1987). As a result of the chelate ring formation the N(am)—Cu—N(im) angle of 85.27 (8)° is significantly smaller than the tetrahedral angle leading to appreciable distortion of the tetrahedral geometry and a large N(im)—Cu—Br angle of 132.01 (6)°. The N1/C7/C6/N2 torsion angle is 58.7 (3)°. The thiophene ring and imine group are near planar, with the sulfur oriented towards the copper atoms. However, Cu—S distances of 3.20 (6) Å make interactions unlikely. The Cu_2_Br_2_ bridging unit forms a planar rhomboid arrangement, with an inversion center in the center. Related structures with a Cu_2_Br_2_ core are published (Ball *et al.*, 2001; Skelton *et al.*, 1991; Churchill, *et al.*, 1984). Cu1 possesses one short (2.4241 (4) Å) and one long (2.4805 (4) Å) Cu–Br bond, and a Cu–Cu distance of 2.980 (0) Å, outside the sum of the van der Waals radii of copper. The arrangement of the bromide bridging unit is asymmetrical: the Cu–Br–Cu bridging angle is 74.829 (13)°, close to the mean value of 74.(9)° found in structural units of this kind in the Cambridge Structural Database (Bruno *et al.*, 2002). Weak C—H···*Cg*π-ring intermolecular interactions contribute to molecular packing in the crystal (Table 1, Fig. 2).

### Experimental

The title compound was synthesized under a dinitrogen atmosphere by reacting a light green suspension of 233 mg of CuBr (1.6 mmol) in 8 mL of dry acetonitrile with 332 mg of the ligand *N*,*N*-dimethyl-*N*'-(thiophen-2-ylmethylene)ethane-1,2-diamine (*L*, 4.8 mmol) added dropwise with a pipet. Addition of ligand resulted in an immediate color change of the solution to red-orange and dissolution of CuBr. The reaction mixture was allowed to stir overnight and filtered. The filtrate was layered with diethyl ether and stored at -25 °C for 4 days. After this time, the product was isolated as orange crystals suitable for X-ray analysis. A second crop was obtained by further addition of diethyl ether and storage at -25°C for 4 days to yield a combined crop of 440 mg of crystalline product (84% yield).

### Refinement

All of the H atoms were placed in their calculated positions and then refined using the riding model with C—H lengths of 0.93 Å (CH), 0.97 Å (CH_2_) or 0.96 Å (CH_3_). The isotropic displacement parameters for these atoms were set from 1.19 to 1.22 (CH, CH_2_), or 1.49 10 to 1.53 (CH_3_) times *U*_eq_ of the parent atom.

### Crystal data

**Table:**

[Cu_2_Br_2_(C_9_H_14_N_2_S)_2_]	*F*(000) = 648
*M**_r_* = 651.48	*D*_x_ = 1.803 Mg m^−^^3^
Monoclinic, *P*2_1_/*c*	Mo *K*α radiation, λ = 0.71073 Å
Hall symbol: -P 2ybc	Cell parameters from 4548 reflections
*a* = 10.2029 (3) Å	θ = 3.1–32.2°
*b* = 15.4175 (3) Å	µ = 5.29 mm^−^^1^
*c* = 8.04875 (19) Å	*T* = 173 K
β = 108.628 (3)°	Rod, red
*V* = 1199.76 (5) Å^3^	0.15 × 0.07 × 0.05 mm
*Z* = 2	

### Data collection

**Table:**

Oxford Diffraction Xcalibur Eos Gemini diffractometer	3928 independent reflections
Radiation source: Enhance (Mo) X-ray Source	3021 reflections with *I* > 2σ(*I*)
Graphite monochromator	*R*_int_ = 0.046
Detector resolution: 16.1500 pixels mm^-1^	θ_max_ = 32.2°, θ_min_ = 3.1°
ω scans	*h* = −15→14
Absorption correction: multi-scan (*CrysAlis PRO*; Oxford Diffraction, 2010)	*k* = −17→22
*T*_min_ = 0.406, *T*_max_ = 1.000	*l* = −11→11
13662 measured reflections	

### Refinement

**Table:**

Refinement on *F*^2^	Secondary atom site location: difference Fourier map
Least-squares matrix: full	Hydrogen site location: inferred from neighbouring sites
*R*[*F*^2^ > 2σ(*F*^2^)] = 0.036	H-atom parameters constrained
*wR*(*F*^2^) = 0.078	*w* = 1/[σ^2^(*F*_o_^2^) + (0.0303*P*)^2^] where *P* = (*F*_o_^2^ + 2*F*_c_^2^)/3
*S* = 1.05	(Δ/σ)_max_ = 0.002
3928 reflections	Δρ_max_ = 0.50 e Å^−^^3^
146 parameters	Δρ_min_ = −0.51 e Å^−^^3^
10 restraints	Extinction correction: *SHELXL97* (Sheldrick, 2008), Fc^*^=kFc[1+0.001xFc^2^λ^3^/sin(2θ)]^-1/4^
Primary atom site location: structure-invariant direct methods	Extinction coefficient: 0.0021 (6)

### Special details

**Table:**

Experimental. ^1^H-NMR (CD_3_CN, 298 K): δ 8.57 (s, 1H, N=CH), 7.64 (m, 2H, thiophene H3, H5), 7.13 (t, J = 4.4 Hz, 1H, thiophene H4), 3.77 (t, J = 5.1 Hz, 2H, NCH_2_), 2.28 (t, J = 5.5 Hz, 2H, NCH_2_), 2.39 (s, 6H, NCH_3_) p.p.m.. ^13^C-NMR (CD_3_CN, 298 K): δ 158.11 (C=N), 141.40 (thiophene C2), 135.94 (thiophene C1 or C3), 132.90 (thiophene C1 or C3), 129.16 (thiophene C4), 61.17 (NCH_2_), 59.33 (NCH_2_), 47.69 (NCH_3_) p.p.m.. FTIR (cm^-1^): 3200 (w), 3073 (*m*), 2989 (*versus*), 2855 (*versus*), 2822 (*versus*), 2779 (*versus*), 1810 (w), 1611 (*versus*), 1452 (*versus*), 1430 (*versus*), 1262 (*s*), 1249 (*s*), 1046 (*s*), 1027 (*s*), 885 (*s*), 713 (*versus*). ESI-MS: m/*z* 427 ([(*L*)_2_Cu]+), m/*z* 245 ([(*L*)Cu]+).
Geometry. All e.s.d.'s (except the e.s.d. in the dihedral angle between two l.s. planes) are estimated using the full covariance matrix. The cell e.s.d.'s are taken into account individually in the estimation of e.s.d.'s in distances, angles and torsion angles; correlations between e.s.d.'s in cell parameters are only used when they are defined by crystal symmetry. An approximate (isotropic) treatment of cell e.s.d.'s is used for estimating e.s.d.'s involving l.s. planes.
Refinement. Refinement of *F*^2^ against ALL reflections. The weighted *R*-factor *wR* and goodness of fit *S* are based on *F*^2^, conventional *R*-factors *R* are based on *F*, with *F* set to zero for negative *F*^2^. The threshold expression of *F*^2^ > σ(*F*^2^) is used only for calculating *R*-factors(gt) *etc*. and is not relevant to the choice of reflections for refinement. *R*-factors based on *F*^2^ are statistically about twice as large as those based on *F*, and *R*- factors based on ALL data will be even larger.

### Fractional atomic coordinates and isotropic or equivalent isotropic displacement parameters (Å^2^)

**Table:**

	*x*	*y*	*z*	*U*_iso_*/*U*_eq_	Occ. (<1)
Br1	0.43830 (3)	0.598179 (15)	0.60809 (3)	0.03225 (9)	
Cu1	0.48455 (3)	0.550941 (19)	0.33751 (4)	0.03010 (10)	
N1	0.2808 (2)	0.54727 (13)	0.1227 (3)	0.0291 (4)	
N2	0.5330 (2)	0.64590 (12)	0.1965 (3)	0.0272 (4)	
S1A	0.8104 (2)	0.58936 (10)	0.4729 (3)	0.0405 (4)	0.719 (3)
C1A	0.7761 (7)	0.6723 (9)	0.3259 (10)	0.0267 (13)	0.719 (3)
C2A	0.8929 (7)	0.7219 (5)	0.3231 (9)	0.0410 (15)	0.719 (3)
H2AA	0.8950	0.7688	0.2511	0.049*	0.719 (3)
C3A	1.0107 (7)	0.6822 (3)	0.4607 (8)	0.0465 (13)	0.719 (3)
H3AA	1.0999	0.7042	0.4885	0.056*	0.719 (3)
C4A	0.9811 (5)	0.6116 (4)	0.5442 (7)	0.0396 (13)	0.719 (3)
H4AA	1.0466	0.5798	0.6295	0.048*	0.719 (3)
S1B	0.9238 (5)	0.7208 (4)	0.3547 (7)	0.0405 (4)	0.281 (3)
C1B	0.7753 (17)	0.670 (3)	0.357 (4)	0.0267 (13)	0.281 (3)
C2B	0.797 (2)	0.6021 (13)	0.484 (3)	0.0410 (15)	0.281 (3)
H2BA	0.7309	0.5676	0.5099	0.049*	0.281 (3)
C3B	0.9481 (16)	0.6001 (12)	0.563 (3)	0.0465 (13)	0.281 (3)
H3BA	0.9903	0.5596	0.6488	0.056*	0.281 (3)
C4B	1.0253 (17)	0.6584 (10)	0.5075 (19)	0.0396 (13)	0.281 (3)
H4BA	1.1212	0.6624	0.5501	0.048*	0.281 (3)
C5	0.6420 (3)	0.68954 (16)	0.2103 (3)	0.0308 (5)	
H5A	0.6323	0.7374	0.1370	0.037*	
C6	0.4062 (3)	0.67187 (17)	0.0565 (4)	0.0376 (6)	
H6A	0.4305	0.7003	−0.0372	0.045*	
H6B	0.3541	0.7126	0.1026	0.045*	
C7	0.3187 (3)	0.59292 (18)	−0.0144 (3)	0.0373 (6)	
H7A	0.2353	0.6106	−0.1061	0.045*	
H7B	0.3695	0.5538	−0.0659	0.045*	
C8	0.1727 (3)	0.59356 (19)	0.1696 (4)	0.0441 (7)	
H8A	0.0927	0.5997	0.0675	0.066*	
H8B	0.2060	0.6499	0.2143	0.066*	
H8C	0.1484	0.5615	0.2577	0.066*	
C9	0.2323 (3)	0.45972 (18)	0.0614 (4)	0.0440 (7)	
H9A	0.1555	0.4636	−0.0449	0.066*	
H9B	0.2036	0.4305	0.1494	0.066*	
H9C	0.3059	0.4278	0.0398	0.066*	

### Atomic displacement parameters (Å^2^)

**Table:**

	*U*^11^	*U*^22^	*U*^33^	*U*^12^	*U*^13^	*U*^23^
Br1	0.04731 (18)	0.02362 (13)	0.02861 (14)	0.00286 (10)	0.01606 (11)	0.00094 (9)
Cu1	0.03302 (19)	0.02855 (17)	0.02764 (16)	−0.00042 (12)	0.00816 (13)	0.00348 (11)
N1	0.0276 (11)	0.0331 (11)	0.0266 (10)	−0.0016 (9)	0.0086 (8)	−0.0014 (8)
N2	0.0286 (11)	0.0240 (10)	0.0284 (10)	0.0017 (8)	0.0085 (8)	0.0006 (8)
S1A	0.0361 (7)	0.0372 (7)	0.0417 (7)	−0.0028 (5)	0.0033 (5)	0.0064 (6)
C1A	0.0332 (14)	0.0278 (14)	0.024 (4)	−0.0043 (10)	0.0161 (16)	−0.005 (3)
C2A	0.030 (3)	0.054 (3)	0.034 (3)	0.001 (2)	0.004 (2)	0.001 (2)
C3A	0.033 (3)	0.048 (3)	0.064 (4)	−0.010 (2)	0.023 (3)	−0.021 (2)
C4A	0.022 (2)	0.045 (3)	0.044 (2)	0.002 (2)	−0.0012 (19)	−0.010 (2)
S1B	0.0361 (7)	0.0372 (7)	0.0417 (7)	−0.0028 (5)	0.0033 (5)	0.0064 (6)
C1B	0.0332 (14)	0.0278 (14)	0.024 (4)	−0.0043 (10)	0.0161 (16)	−0.005 (3)
C2B	0.030 (3)	0.054 (3)	0.034 (3)	0.001 (2)	0.004 (2)	0.001 (2)
C3B	0.033 (3)	0.048 (3)	0.064 (4)	−0.010 (2)	0.023 (3)	−0.021 (2)
C4B	0.022 (2)	0.045 (3)	0.044 (2)	0.002 (2)	−0.0012 (19)	−0.010 (2)
C5	0.0361 (15)	0.0243 (12)	0.0340 (13)	−0.0020 (10)	0.0140 (11)	0.0022 (9)
C6	0.0387 (16)	0.0341 (14)	0.0357 (14)	−0.0003 (11)	0.0057 (11)	0.0111 (11)
C7	0.0346 (15)	0.0486 (16)	0.0265 (13)	−0.0048 (12)	0.0068 (11)	0.0045 (11)
C8	0.0316 (16)	0.0579 (18)	0.0443 (16)	0.0077 (13)	0.0140 (13)	0.0023 (13)
C9	0.0428 (17)	0.0437 (16)	0.0417 (16)	−0.0137 (13)	0.0084 (13)	−0.0083 (12)

### Geometric parameters (Å, º)

**Table:**

Br1—Cu1^i^	2.4241 (4)	S1B—C1B	1.71 (2)
Br1—Cu1	2.4805 (4)	C1B—C2B	1.43 (2)
Cu1—N2	2.008 (2)	C1B—C5	1.521 (18)
Cu1—N1	2.240 (2)	C2B—C3B	1.473 (19)
Cu1—Br1^i^	2.4242 (4)	C2B—H2BA	0.9300
Cu1—Cu1^i^	2.9803 (6)	C3B—C4B	1.360 (14)
N1—C8	1.460 (3)	C3B—H3BA	0.9300
N1—C7	1.462 (3)	C4B—H4BA	0.9300
N1—C9	1.468 (3)	C5—H5A	0.9300
N2—C5	1.274 (3)	C6—C7	1.509 (4)
N2—C6	1.474 (3)	C6—H6A	0.9700
S1A—C4A	1.685 (5)	C6—H6B	0.9700
S1A—C1A	1.701 (9)	C7—H7A	0.9700
C1A—C5	1.413 (7)	C7—H7B	0.9700
C1A—C2A	1.422 (11)	C8—H8A	0.9600
C2A—C3A	1.482 (8)	C8—H8B	0.9600
C2A—H2AA	0.9300	C8—H8C	0.9600
C3A—C4A	1.364 (7)	C9—H9A	0.9600
C3A—H3AA	0.9300	C9—H9B	0.9600
C4A—H4AA	0.9300	C9—H9C	0.9600
S1B—C4B	1.641 (11)		
			
Cu1^i^—Br1—Cu1	74.829 (13)	C1B—C2B—H2BA	128.6
N2—Cu1—N1	85.27 (8)	C3B—C2B—H2BA	128.6
N2—Cu1—Br1^i^	132.01 (6)	C4B—C3B—C2B	118.9 (18)
N1—Cu1—Br1^i^	106.45 (5)	C4B—C3B—H3BA	120.5
N2—Cu1—Br1	115.60 (6)	C2B—C3B—H3BA	120.5
N1—Cu1—Br1	107.16 (6)	C3B—C4B—S1B	109.8 (15)
Br1^i^—Cu1—Br1	105.171 (13)	C3B—C4B—H4BA	125.1
N2—Cu1—Cu1^i^	154.69 (6)	S1B—C4B—H4BA	125.1
N1—Cu1—Cu1^i^	118.42 (5)	N2—C5—C1A	126.4 (5)
Br1^i^—Cu1—Cu1^i^	53.447 (11)	N2—C5—C1B	120.1 (10)
Br1—Cu1—Cu1^i^	51.724 (11)	N2—C5—H5A	116.8
C8—N1—C7	111.3 (2)	C1A—C5—H5A	116.8
C8—N1—C9	109.6 (2)	C1B—C5—H5A	122.5
C7—N1—C9	109.3 (2)	N2—C6—C7	109.8 (2)
C8—N1—Cu1	112.30 (16)	N2—C6—H6A	109.7
C7—N1—Cu1	99.58 (15)	C7—C6—H6A	109.7
C9—N1—Cu1	114.39 (16)	N2—C6—H6B	109.7
C5—N2—C6	116.8 (2)	C7—C6—H6B	109.7
C5—N2—Cu1	134.54 (17)	H6A—C6—H6B	108.2
C6—N2—Cu1	108.42 (15)	N1—C7—C6	111.7 (2)
C4A—S1A—C1A	92.6 (3)	N1—C7—H7A	109.3
C5—C1A—C2A	121.9 (7)	C6—C7—H7A	109.3
C5—C1A—S1A	122.7 (6)	N1—C7—H7B	109.3
C2A—C1A—S1A	115.4 (5)	C6—C7—H7B	109.3
C1A—C2A—C3A	104.4 (6)	H7A—C7—H7B	107.9
C1A—C2A—H2AA	127.8	N1—C8—H8A	109.5
C3A—C2A—H2AA	127.8	N1—C8—H8B	109.5
C4A—C3A—C2A	116.4 (6)	H8A—C8—H8B	109.5
C4A—C3A—H3AA	121.8	N1—C8—H8C	109.5
C2A—C3A—H3AA	121.8	H8A—C8—H8C	109.5
C3A—C4A—S1A	111.2 (5)	H8B—C8—H8C	109.5
C3A—C4A—H4AA	124.4	N1—C9—H9A	109.5
S1A—C4A—H4AA	124.4	N1—C9—H9B	109.5
C4B—S1B—C1B	94.1 (10)	H9A—C9—H9B	109.5
C2B—C1B—C5	126 (2)	N1—C9—H9C	109.5
C2B—C1B—S1B	114.3 (12)	H9A—C9—H9C	109.5
C5—C1B—S1B	118.4 (15)	H9B—C9—H9C	109.5
C1B—C2B—C3B	102.8 (15)		
			
Cu1^i^—Br1—Cu1—N2	−154.00 (7)	C2A—C3A—C4A—S1A	−2.6 (7)
Cu1^i^—Br1—Cu1—N1	113.00 (6)	C1A—S1A—C4A—C3A	1.5 (6)
Cu1^i^—Br1—Cu1—Br1^i^	0.0	C4B—S1B—C1B—C2B	2 (3)
N2—Cu1—N1—C8	−100.19 (18)	C4B—S1B—C1B—C5	−168 (2)
Br1^i^—Cu1—N1—C8	127.30 (16)	C5—C1B—C2B—C3B	167 (3)
Br1—Cu1—N1—C8	15.16 (18)	S1B—C1B—C2B—C3B	−2 (3)
Cu1^i^—Cu1—N1—C8	70.41 (18)	C1B—C2B—C3B—C4B	2 (3)
N2—Cu1—N1—C7	17.69 (15)	C2B—C3B—C4B—S1B	0 (2)
Br1^i^—Cu1—N1—C7	−114.82 (14)	C1B—S1B—C4B—C3B	−0.8 (19)
Br1—Cu1—N1—C7	133.04 (14)	C6—N2—C5—C1A	−175.2 (7)
Cu1^i^—Cu1—N1—C7	−171.71 (13)	Cu1—N2—C5—C1A	10.8 (7)
N2—Cu1—N1—C9	134.13 (18)	C6—N2—C5—C1B	176.8 (17)
Br1^i^—Cu1—N1—C9	1.62 (18)	Cu1—N2—C5—C1B	2.8 (18)
Br1—Cu1—N1—C9	−110.51 (17)	C2A—C1A—C5—N2	175.9 (7)
Cu1^i^—Cu1—N1—C9	−55.26 (19)	S1A—C1A—C5—N2	−2.0 (13)
N1—Cu1—N2—C5	−174.8 (2)	C2A—C1A—C5—C1B	−135 (14)
Br1^i^—Cu1—N2—C5	−66.8 (3)	S1A—C1A—C5—C1B	47 (12)
Br1—Cu1—N2—C5	78.5 (2)	C2B—C1B—C5—N2	4 (4)
Cu1^i^—Cu1—N2—C5	24.9 (3)	S1B—C1B—C5—N2	172.0 (16)
N1—Cu1—N2—C6	10.83 (16)	C2B—C1B—C5—C1A	−132 (16)
Br1^i^—Cu1—N2—C6	118.76 (15)	S1B—C1B—C5—C1A	37 (11)
Br1—Cu1—N2—C6	−95.95 (16)	C5—N2—C6—C7	146.5 (2)
Cu1^i^—Cu1—N2—C6	−149.55 (14)	Cu1—N2—C6—C7	−38.0 (3)
C4A—S1A—C1A—C5	177.9 (9)	C8—N1—C7—C6	75.1 (3)
C4A—S1A—C1A—C2A	−0.2 (9)	C9—N1—C7—C6	−163.7 (2)
C5—C1A—C2A—C3A	−179.2 (9)	Cu1—N1—C7—C6	−43.5 (2)
S1A—C1A—C2A—C3A	−1.1 (11)	N2—C6—C7—N1	58.7 (3)
C1A—C2A—C3A—C4A	2.3 (9)		

Symmetry code: (i) −*x*+1, −*y*+1, −*z*+1.

### Hydrogen-bond geometry (Å, º)

Cg1, Cg3 and Cg4 are the centroids of the Br1/Cu1/Br1*A*/Cu1*A*, S1*A*/C1*A*/C2*A*/C3*A*/C4*A* and S1*B*/C1*B*/C2*B*/C3*B*/C4*B* rings, respectively.

**Table:**

*D*—H···*A*	*D*—H	H···*A*	*D*···*A*	*D*—H···*A*
C2*A*—H2*AA*···*Cg*3^ii^	0.93	2.87	3.721 (8)	153
C2*A*—H2*AA*···*Cg*4^ii^	0.93	2.70	3.573 (12)	157
C2*B*—H2*BA*···*Cg*1	0.93	2.55	3.45 (2)	162
C2*B*—H2*BA*···*Cg*1^i^	0.93	2.55	3.45 (2)	162

Symmetry codes: (i) −*x*+1, −*y*+1, −*z*+1; (ii) *x*, −*y*+1/2, *z*−3/2.
